# *In Vivo* Antioxidant and Anti-Skin-Aging Activities of Ethyl Acetate Extraction from *Idesia polycarpa* Defatted Fruit Residue in Aging Mice Induced by D-Galactose

**DOI:** 10.1155/2014/185716

**Published:** 2014-05-25

**Authors:** Yang Ye, Ran-ran Jia, Lin Tang, Fang Chen

**Affiliations:** National and Local Joint Engineering Laboratory for Energe Plant Bio-Oil Production and Application, Sichuan Key Laboratory of Resource Biology and Biopharmaceutical Engineering, Key Laboratory of Bio-Resources and Eco-Environment, Ministry of Education, College of Life Sciences, Sichuan University, Chengdu, Sichuan 610064, China

## Abstract

Two different concentrations of D-galactose (D-gal) induced organism and skin aging in Kunming mice were used to examine comprehensively the antioxidant and antiaging activities of ethyl acetate extraction (EAE) from *Idesia polycarpa* defatted fruit residue for the first time. The oxygen radical absorbance capacity (ORAC) of EAE was 13.09 ± 0.11 **μ**mol Trolox equivalents (TE)/mg, which showed EAE had great *in vitro* free radical scavenging and antioxidant activity. Biochemical indexes and morphological analysis of all tested tissues showed that EAE could effectively improve the total antioxidant capacity (T-AOC) of the antioxidant defense system of the aging mice, enhance the activities of superoxide dismutase (SOD), catalase (CAT), and glutathione peroxidase (GSH-Px) of tissues and serum, increase glutathione (GSH) content and decrease the malondialdehyde (MDA) content, and maintain the skin collagen, elastin, and moisture content. Meanwhile, EAE could effectively attenuate the morphological damage in brain, liver, kidney, and skin induced by D-gal and its effect was not less than that of the well-known L-ascorbic acid (VC) and **α**-tocopherol (VE). Overall, EAE is a potent natural antiaging agent with great antioxidant activity, which can be developed as a new medicine and cosmetic for the treatment of age-related conditions.

## 1. Introduction

One of the reasons why people will be senile gradually is the oxidative stress. Oxidative stress is a result of imbalance between the formation of reactive oxygen species (ROS) and the* in vivo* antioxidant system [1, 2]. ROS contain oxygen free radicals and hydrogen peroxide [3]. Excess ROS can make the lipids of blood, cells, tissues, and so on into lipid peroxides. These peroxides can deposit on the cell membrane, which will result in the loss of function of the cell membrane and decrease in cell viability. Furthermore, the function of tissues and organs will decline and then the human body will be sped up to get senile. In addition, ROS can cause cell damage, death, and exacerbate several age-related chronic diseases including cancer, Alzheimer's disease, Parkinson's disease, and heart disease [4]. More and more studies show that antioxidant is critical to prevent aging.

Skin aging is part of the major signs of bodily aging. Genetics, environmental exposure, hormonal changes, and metabolic processes influence the skin ageing process [5]. Hence, it is believed that intrinsic and extrinsic damage are the primary causes of skin aging [6]. The skin uses a series of intrinsic antioxidants to be protected from free radical damage. Naturally, increasing extrinsic antioxidants have also been widely exhibiting skin anti-aging activity [2]. Therefore, antioxidants are considered as important nutraceuticals with healthy benefits and additives of antiaging cosmetics.

Current aging models of rats, mice, and other rodents mainly include aging animal model induced by D-gal [7], senescence accelerated mouse (SAMP) model [8], and natural aging model. Compared with the rest of animal aging models, the modelling method of D-gal animal model is simple, inexpensive, and stable [9, 10]. D-gal animal model has become an internationally recognized animal model and has been widely used in the field of antiaging medicines. Animals are injected D-gal continuously within a certain time, which will greatly increase the concentration of intracellular galactose* in vivo*. The galactose is reduced to galactitol in the catalysis of aldose reductase; galactitol cannot be further metabolized and thus accumulate in the cell, which will change the normal osmotic pressure, so that leads to cell swelling and dysfunction and ultimately leads to aging [11]. In addition, free radicals generated from D-gal metabolism* in vivo* can result in aging as well [4, 12]. Therefore, using D-gal to build the body or the skin-aging model is both reasonable and effective.

*Idesia polycarpa* is a deciduous tree of the Flacourtiaceae family and distributes widely. The oil of* Idesia polycarpa* fruit is rich in various unsaturated fatty acids, such as linoleic acid (the linoleic acid content reaches up to 58%–81% of* Idesia polycarpa* oil) [13] and linolenic acid, which has been testified nontoxicity and produced cooking oil in Asia for many years [14]. However, fruit residue does not get a reasonable utilization after oil extraction. There are many phenolics with various kinds of biological activity in defatted residue. As in our previous researches, the EAE from the crude extract of total phenolics of* Idesia polycarpa* has very significant* in vitro* antioxidant, inhibition of tyrosinase, and skin-whitening activity. In addition, idescarpin with whitening activity [15], salirepin, idesin, idesin hydrogen sulfate, and so on [16] separated and identified from EAE still have anti-inflammatory effect [17, 18].

As far as we know, there are no reports on the* in vivo* antioxidant and antiaging activity of phenolics extracted from* Idesia polycarpa* defatted fruit residue. Then, D-gal induced aging Kunming mice were used to examine the antioxidant and antiaging activities of EAE. We used two different concentrations of D-gal to induce organism and skin aging in Kunming mice for 42 d; 100, 200, and 400 mg/kg of EAE were orally administrated once daily in deferring-organism-aging experiment, while 0.5 mL of 1%, 2%, and 4% of EAE was smeared twice daily in deferring-skin-aging experiment. At the end of the experiment, organ indexes, biochemical indicators for antioxidant activity, and histological characteristics were determined. Therefore, this study will lay the foundation for development and utilization of* Idesia polycarpa *in delaying organism and skin aging in mice caused by D-gal.

## 2. Material and Methods

### 2.1. Fruit Samples

Fruits of* Idesia polycarpa* were collected from Guangyuan (Sichuan, China). After being placed in the shade to dry up to constant weight at room temperature, fruits were ground and sieved with a 40-mesh sieve. Before analysis, these samples effectively removed surplus oil with petroleum ether for 5 h by using a Soxhlet system. The defatted powder (1000 g) was extracted with 50 mL 50% ethanol at 80°C for 5 h according to our previous researches. Then, the crude extract was filtered and concentrated in a rotary evaporator to remove solvent. The yield of the product was 40.6% and the product was labelled the crude extract (CE). CE was dissolved in redistilled water in preference to fractional extraction using ethyl acetate. The mixture was left to stand until two phases could be distinguished clearly and the ethyl acetate phase was collected. Each extraction was performed three times under the same conditions; organic phases were concentrated by reducing pressure and dried by vacuum. The yield of ethyl acetate extraction (EAE) was 7.1% of dry powder. The phenolics and flavonoids contents of EAE were 184.17 ± 6.37 of mgGAE/g (mg Gallic acid equivalent/g of EAE extracted) and 168.12 ± 6.00 of mgCaE/g (mg catechin equivalent/g of EAE extracted) according to our previous researches. EAE was stockpiled at −20°C.

### 2.2. Oxygen Radical Absorbance Capacity (ORAC)

The ORAC value of EAE was determined using the method of Alañón, et al. [19] with some modifications. 40 *μ*L of different concentrations (25–200 *μ*M) of Trolox dilutions, 40 *μ*L of EAE (20 *μ*g/mL), or 40 *μ*L of phosphate buffer was pipetted in triplicate for the standard curve, sample, or blank, respectively. Then 40 *μ*L of the fluorescein solution (40 nM) was added. The microplate was incubated at 37°C for 20 min. 170 *μ*L of 2,2′-azobis-2-methyl-propanimidamide dihydrochloride (AAPH, 100 mM) was added and the fluorescence was measured at an excitation wavelength of 485 nm and an emission wavelength of 538 nm every 3 min thereafter 180 min. Calculations were based on the area under the fluorescence decay curve (AUC). ORAC values were calculated using a regression equation for a linear regression on Trolox standards. The net area under the curve was calculated by subtracting the area under the curve for the blank values from the curves of samples and standards. ORAC values were expressed in *μ*mol Trolox equivalents per mg (*μ*mol TE/mg).

### 2.3. Animals and Treatment

All experiments were subject to approval by the Institutional Animal Care and Use Committee of Sichuan University. Six-week-old Kunming mice, weighing around 30.0 ± 2.0 g, were obtained from Dashou biological technology Co., LTD., Chengdu, China. Throughout the experiment, animals were housed in an air-conditioned room, temperature maintained at 25–27°C under daylight cycle of 12 h. The animals were acclimatized for 7 d prior to the experiment. In the process of experimenting, the daily performance and body weight of mice were recorded every day.

In deferring-organism-aging experiment, all mice were split into six groups at random; each group had ten mice with five male mice and female mice. Model group, VC group, and EAE groups were subcutaneously injected with D-gal prepared in normal saline once daily at a dose of 100 mg/kg per day for 42 d, while mice in the control group were treated with the same volume of normal saline. After injection of D-gal, VC group mice were intragastrically administrated with VC at the dose of 100 mg/kg per day and EAE groups were treated with EAE by using intragastric administration at the doses of 100, 200, and 400 mg/kg per day, respectively. Meanwhile, the model and control group were orally administrated the same volume of normal saline for 42 d. At the last treatment, mice were sacrificed on the second day; then blood and tissues were immediately collected, weighed, and homogenized (4°C, 3000 r/min for 10 min) for biochemical and histological analyses. The organ index was measured by using the following equation: organ index (mg/g) = (organ weight (mg)/body weight (g)) [20].

In deferring-skin-aging experiment, as previously noted, 60 male mice were divided into six groups. After shaving their hair, model group, VE group, and EAE groups were subcutaneously injected with D-gal prepared in normal saline once daily at a dose of 1000 mg/kg per day for 42 d, while mice in the control group were treated with the same volume of normal saline. 1%, 2%, 4% EAE, or 1% VE were prepared in water/propylene glycol/ethanol (6 : 3 : 1 v/v/v) with 1% menthol. Each group was smeared twice daily with 0.5 mL water/propylene glycol/ethanol with 1% menthol (control and model group), 1% EAE, 2% EAE, 4% EAE, or 1% VE, respectively. 1 h after the final treatment, mice were sacrificed. Then skins were immediately collected and homogenized (4°C, 3000 r/min for 10 min) for biochemical and histological analyses.

### 2.4. Biochemical Analysis and Histological Analysis

The levels of GSH, hydroxyproline (Hyp), MDA, and total protein and the activities of total SOD, CAT, T-AOC, and GSH-Px in blood and tissues were determined by using relevant commercial kits (Nanjing Jiancheng Bioengineering Institute, China). The content of elastin was determined by utilizing relevant commercial mouse elastin Elisa Kit (Shanghai Bangyi Trading Co., LTD., China).

After being fixed in 10% formalin for 24 h, tissues and dorsal skin samples were progressively dehydrated in different concentrations of ethanol, hyalinized in xylene, embedded in paraffin, sliced into thin sections (5 *μ*m), dewaxed, and colored with hematoxylin-eosin (HE). Cross sections were selected from three plates per sample. The thickness of the epidermis and dermis was measured by using a digimatic caliper (Mitutoyo Co., Kanagawa, Japan).

### 2.5. Statistical Analysis

Each test was performed from 10 mice and all results were expressed as the means plus-minus their corresponding standard deviation (SD). Statistical analysis was measured by using the SPSS software (SPSS, IBM, version 19). The significance of difference among the means was determined by using one way ANOVA. A value of *P* < 0.05 was considered to be statistically significant.

## 3. Results

The ORAC of EAE was 13.09 ± 0.11 *μ*mol TE/mg.

### 3.1. Behavior Observations and Organ Index

Both of the animal experiments had no significant differences in food intake and body weight change (data is not shown). Before they were sacrificed, no mice died during the completely experimental process. Mice of model group with D-gal showed obvious symptoms of aging such as listlessness, lag in response, slow movement, and withered and lackluster fur. In addition, organ indexes of the brain, liver, and kidney of the model group were significantly lower than those of the control group as shown in Table 1 (*P* < 0.01, resp.). EAE and VC could improve those indexes (*P* < 0.05 or *P* < 0.01, resp.). All organ indexes of the high dose EAE group have no significant differences to those of the control group (*P* > 0.05).

### 3.2. Effect of EAE on the MDA Content in Aging Mice

**Figure 1:**
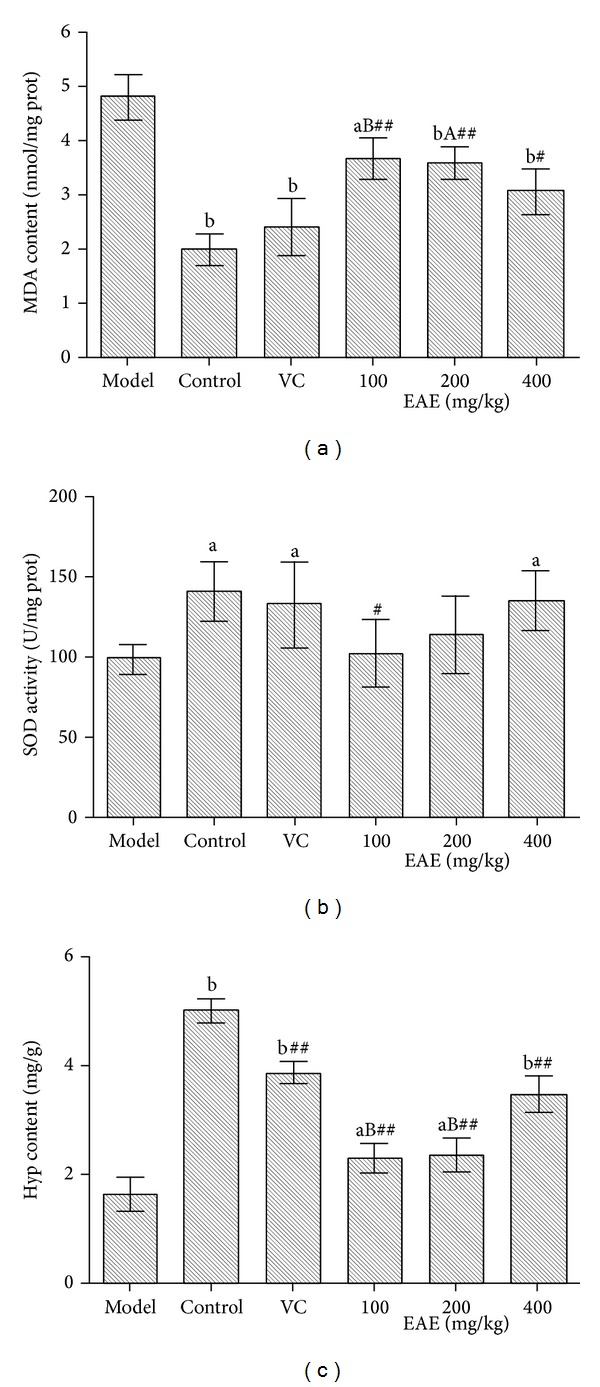
Biochemical indexes of mice skin induced by D-gal in deferring-organism-aging experiment.(a) The effects of EAE on the changes of MDA content, (b) the effects of EAE on the changes of SOD activity, and (c) the effects of EAE on the changes of Hyp content. Each value is the means ± SD (*n* = 10). (a) *P* < 0.05; (b) *P* < 0.01, as compared with the model group (all groups); A: *P* < 0.05; B: *P* < 0.01, as compared with the VC group (EAE groups). ^#^ *P* < 0.05; ^##^ *P* < 0.01 compared with control group (VC and EAE groups).

According to Table 2, the MDA content of model group in the brain was significantly greater than that of control group (*P* < 0.05), which illustrated aging animal model had been set up successfully. The MDA contents of VC group and EAE groups were much smaller than that of model group (*P* < 0.01; low dose EAE group, *P* < 0.05), but the VC group had no significant difference to EAE groups (*P* > 0.05). Especially, the medium and high dose groups were much lower than that of the control group (*P* < 0.05 or *P* < 0.01). As for MDA in the liver, kidney, and serum, EAE groups were lower than model group (*P* < 0.05 or *P* < 0.01). Moreover, the MDA of skin in aging mice has been effectively reduced by intragastric administration of EAE and VC as shown in Figure 1. EAE could reduce the content of MDA in the skin in a dose dependent manner and high dose group had no significant difference to the VC group (*P* > 0.05).

In the deferring-skin-aging experiment, the MDA content of the model group was significantly higher than that of control group (*P* < 0.01), VE group (*P* < 0.01), and EAE groups (*P* < 0.01) as shown in Table 2. In particular, the medium and high dose groups were much smaller than that of control group (*P* < 0.05). As a result of the intragastric experiment, EAE reduced the content of MDA in the skin in a dose dependent manner. Each dose of EAE groups had no significant difference to the VE group (*P* > 0.05).

### 3.3. Effect of EAE on the SOD Activity in Aging Mice

As shown in Table 2, model group in the brain was lower than the control group, VC group, and EAE groups, respectively (*P* < 0.05 or *P* < 0.01). However, VC group had no significant difference to EAE groups (*P* > 0.05). The results of other tissues had the same situation, except that VC group was lower than low dose EAE group (*P* < 0.05), medium dose EAE group (*P* < 0.01), and high dose EAE group (*P* < 0.01) in the kidney. The SOD activities of the skins of control group, VC group, and the high dose group were obviously higher than that of model group (*P* < 0.01), and there were no significant differences among the three groups (*P* > 0.05) as shown in Figure 1.

Compared with the control group, model group decreased significantly in the activity of SOD in deferring-skin-aging experiment (*P* < 0.01). High dose EAE group and VE group were significantly higher than control group (*P* < 0.01); medium and high dose group had no significant difference to the VE group.

### 3.4. Effect of EAE on the GSH Content in Aging Mice

The content of GSH (glutathione) is the key factor for measuring the level of antioxidative activity* in vivo*. It also could be seen in the content of GSH in brain that model group was lower than the control group, VC group, and EAE groups (*P* < 0.05 or *P* < 0.01) as shown in Table 2. As for other tissues, model group was lower than the control group (*P* < 0.05 or *P* < 0.01). In the brain, medium dose EAE group (*P* < 0.01) and high dose EAE group (*P* < 0.01) were significantly higher than the VC group, while the low dose group had no significant difference to the VC group. The VC group did not significantly improve the content of GSH in serum of aging mice (*P* > 0.05), while medium dose group (*P* < 0.05) and high dose group (*P* < 0.01) sharply increased more than model group.

### 3.5. Effect of EAE on the GSH-Px Activity in Aging Mice

The GSH-Px activities of the liver, kidney, and serum of model group were significantly lower than those of control groups, VC groups, and EAE groups as shown in Table 2. Medium dose EAE groups could more effectively enhance the GSH-Px activities than the VC group in liver (*P* < 0.01) and serum (*P* < 0.05), while the high dose group was most effective in kidney. GSH-Px activities of 10% brain homogenates have not been detected.

### 3.6. Effect of EAE on the CAT Activity in Aging Mice

The medium dose group, the high dose group, and the VC group could significantly enhance CAT activities in liver more than the model group (*P* < 0.01); medium and high dose group could effectively improve the activity in the liver of aging mice than control mice (*P* < 0.05 and *P* < 0.01, resp.). Moreover, the high dose group was more effective than the VC group (*P* < 0.01). In the kidney, the medium dose group was the most effective group. However, CAT activity is expected to be very low in the brain; the CAT activities of 10% brain homogenates have not been detected.

### 3.7. Effect of EAE on the T-AOC Activity in Aging Mice

Control groups and EAE groups were considerably higher than model groups in all tissues. VC groups in the liver and kidney had no meaningful difference to model groups (*P* > 0.05), while high dose groups could effectively enhance the T-AOC activity compared to control groups in these two tissues (*P* < 0.01). VC, as the positive control, could maintain availably the T-AOC activity in the serum of aging mice as that of control mice. The T-AOC activities of the serum of low (*P* < 0.05) and medium dose groups (*P* < 0.05) were not as good as that of VC group, but the high dose group had no significant difference to the VC group (*P* > 0.05).

### 3.8. Effect of EAE on the Hyp Content in the Skin of Aging Mice

Hyp content of the old is less than that of the young. As shown in Figure 1, VC and EAE could retain the Hyp content in aging mice, and EAE were in a dose dependent manner. The high dose group had no significant difference to the VC group (*P* > 0.05).

From the results of the smearing experiment, EAE could obviously increase the hydroxyproline content of skin on skin-aging mice, thus promoting skin collagen synthesis. Control group (*P* < 0.01), VE group (*P* < 0.01), and each dose group (*P* < 0.01) were significantly different from the model group as shown in Table 3. Low dose (*P* < 0.01) and medium dose group (*P* < 0.05) were lower than the VE group, but the high dose group had no significant difference to the VE group (*P* > 0.05).

### 3.9. Effect of EAE on the Elastin Content of the Skin of Aging Mice

In our experiment, the loss of elastin caused by modelling showed that model group was much lower than control group (*P* < 0.05), which verified elastin would gradually be consumed with the growth of the age. Although the content of elastin measured by Elisa was low, significant differences among the groups were quite apparent. VE group (*P* < 0.05), medium (*P* < 0.05), and high dose group (*P* < 0.01) were much higher than model group, and there were no differences between the VE group and EAE groups (*P* > 0.05, resp.).

### 3.10. Effect of EAE on the Moisture Content of the Skin of Aging Mice

Even though the medium and high dose groups had no significant difference to the VE group (*P* > 0.05, resp.), high dose EAE (*P* > 0.05) was not as moisturizing, effective as medium dose EAE (*P* < 0.05) and VE (*P* < 0.01) to the aging mouse skin. The moisture content of the model group was significantly lower than that of the control group.

### 3.11. Morphological Alterations

**Figure 2:**
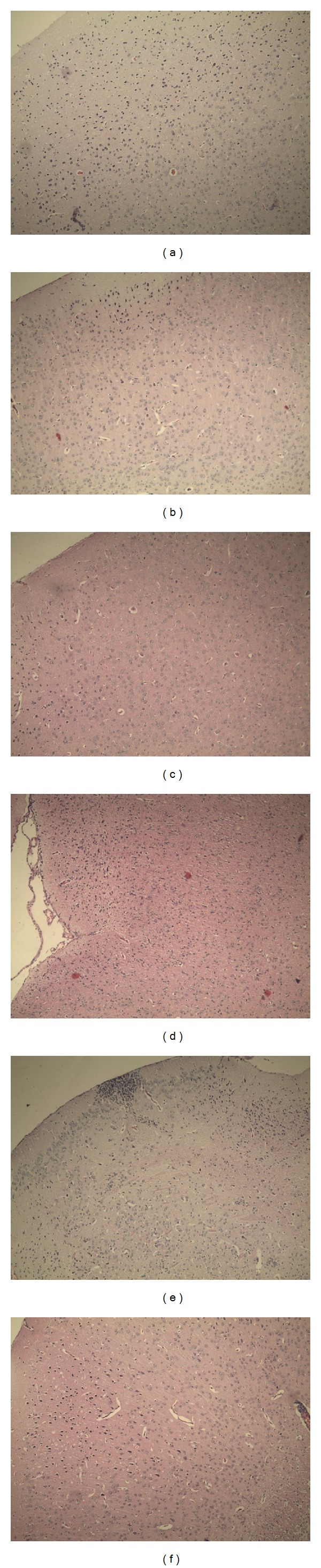
Morphological changes of aging mice brains induced by D-gal in deferring-organism-aging experiment. Hematoxylin and eosin-stained brains of the treated mice. (a) Model group; (b) control group; (c) VC group (100 mg/kg); and (d, e, f) EAE groups (100, 200, and 400 mg/kg). Original magnification, 100x.

We have made the paraffin sections of the brains, the livers, and the kidneys in the deferring-organism-aging experiment and of the skins in the deferring-skin-aging experiment. From Figure 2, the number of brain cells of the model group was less than that of the control, VC, and EAE groups found with the unaided eye. Brain cells will decline with age, so reducing the loss of brain cells is important. VC played a role in protecting brain as the report; the VC group indicated no obvious difference to control group.

**Figure 3:**
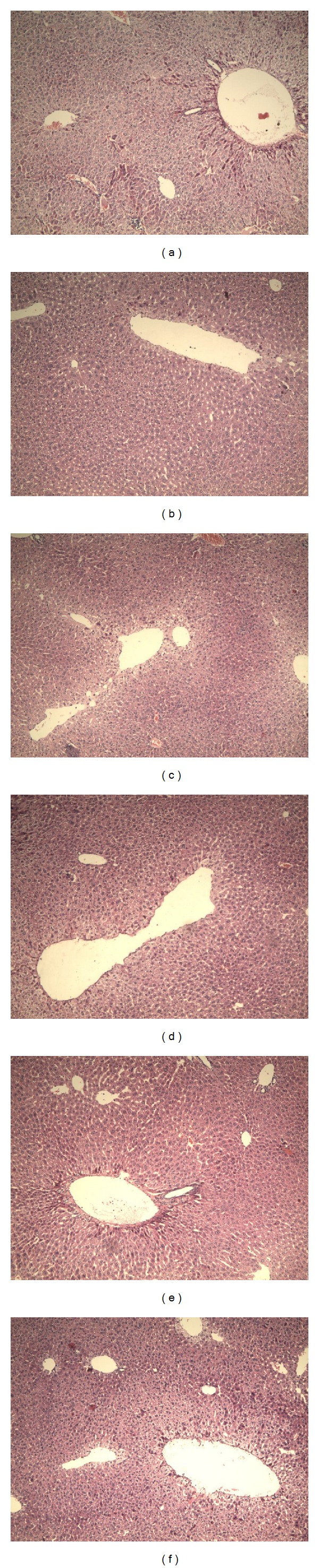
Morphological changes of aging mice livers induced by D-gal in deferring-organism-aging experiment. Hematoxylin and eosin-stained livers of the treated mice. (a) Model group; (b) control group; (c) VC group (100 mg/kg); and (d, e, f) EAE groups (100, 200, and 400 mg/kg). Original magnification, 100x.

**Figure 4:**
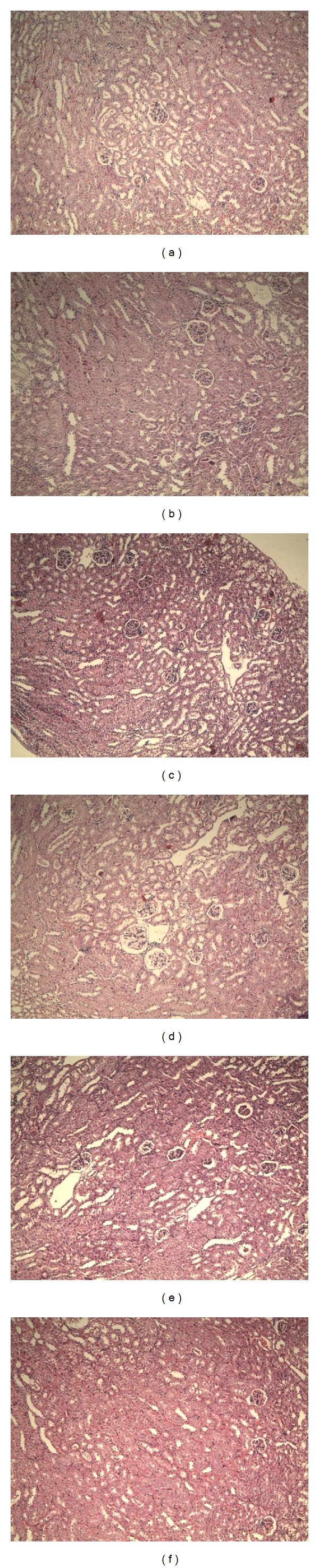
Morphological changes of aging mice kidneys induced by D-gal in deferring-organism-aging experiment. Hematoxylin and eosin-stained kidneys of the treated mice. (a) Model group; (b) control group; (c) VC group (100 mg/kg); and (d, e, f) EAE groups (100, 200, and 400 mg/kg). Original magnification, 100x.

The morphological features of haematoxylin and eosin (H&E) stained liver sections were presented in Figure 3. The hepatocytes of D-gal-treated mice showed an extensive hepatic demo and some degree of ballooning degeneration and the cytoplasm color of hepatocytes became shallow as opposed to that of normal mice. Intriguingly, EAE treatment could attenuate liver injury induced by D-gal in mice. All different concentrations of EAE showed excellent liver-protecting activity; the hepatic edema was largely controlled. Similarly, the renal tubular epithelial cells of D-gal-treated mice showed edema as shown in Figure 4. The cell membrane penetrability of the epithelial cells was a major increase and the cytoplasm color became shallow and even transparent. Medium and high dose EAE could mitigate renal tubular edema thus protecting the kidney.

**Figure 5:**
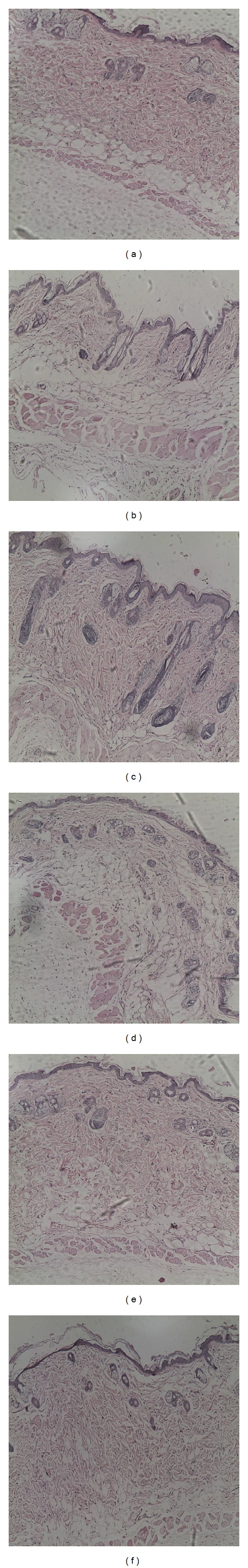
Morphological changes of aging mice skins induced by D-gal in deferring-skin-aging experiment. Hematoxylin and eosin-stained skins of the treated mice. (a) Control group; (b) model group; (c) VE group (1%); and (d, e, f) EAE groups (1%, 2%, and 4%). Original magnification, 100x.

Compared with normal mice, the model mice showed a significant (*P* < 0.05) decrease in the thickness of the epidermis and dermis as shown in Table 4, as well as a decrease in forming of fibroblast as shown in Figure 5, but this change was significantly attenuated by long-term administration of VE or EAE. The number of fibroblast determines the content of collagen fibers, so these results were consistent with the previous results of the hydroxyproline content. EAE could increase the content of fibroblast, thus helping repair damaged skin and reducing the aging effects on the skin.

## 4. Discussion and Conclusion 

From a biological perspective, aging is an inevitable spontaneous process and a complicated natural phenomenon [12, 21]. With the improvement of living standards all over the world, people are increasingly concerned about their appearance and health. How to postpone skin and body aging becomes a hot topic of concern for many people.

With increasing lifespan comes functional decline and atrophy on all tissues and organs. Particularly, changes in brain and kidney weight are obvious [22]. Therefore, change of organ index is an important indicator of organism senescence [20]. D-gal could induce bodily aging and sharply reduce organ indexes of the brain, liver, and kidney in the model group according to our experiment. Compared with the model group, EAE could effectively improve all organ indexes. It illustrated that EAE could play the role of protecting the weight of those organs from descending along with getting older caused by D-gal and had the obvious antiaging effect to the brain, liver, and kidney.

Living in an oxygenated environment has required the evolution of effective cellular strategies to detect and detoxify metabolites of ROS. However or wherever ROS are generated, a rise in intracellular oxidant levels has two potentially important effects: damaging of various cell components and triggering of the activation of specific signalling pathways [1]. These effects can affect significantly a host of physiological processes and metabolic pathway closely bounded up with the aging of the organism and skin or the development of age-related disorders. For instance, excess ROS can make lipid peroxidated* in vivo*. The final oxidation product is MDA. MDA can direct the protein and nucleic acid to cross-link and MDA have cytotoxicity [23]. Therefore, the content of MDA may reflect directly the body's level of lipid peroxidation and reflect indirectly the level of cell damage brought out by ROS.

A complex antioxidant-defense system, including the major enzymatic scavengers SOD, CAT, and GSH-Px, can eliminate most of the adverse impact caused by ROS. SOD can catalyze the rapid transformation of superoxide to hydrogen peroxide [24]. SOD is the primary free radical scavenger* in vivo*, an enzyme whose sole function appeared to be the removal of superoxide anions, and SOD activity is the visual indicator of the level of organism aging [25]. GSH-Px can reduce toxic peroxide into nontoxic hydroxyl compound, thereby preserving the structure and function of cell membrane without interference and damage from peroxide [21]. CAT can decompose H_2_O_2_ into molecular oxygen and water and clear the hydrogen peroxide* in vivo*, so that it can protect cells from the damage of H_2_O_2_ [26]. CAT is found in all known animal organizations, particularly with high concentrations in liver [27].

There are diverse nonenzymatic and low molecular mass molecules, including pyruvate, ascorbate, polyphenol, and flavonoids,, and they can scavenge ROS as well. Glutathione (GSH), one of those molecules, is synthesized from glutamic acid, cysteine, and glycine. GSH can be discovered in almost every cell of the body and have excellent antioxidant and detoxifying activity. GSH cannot only eliminate free radicals* in vivo* but also enhance the organism immunity [28]. As the* in vivo* content of GSH, T-AOC is also an indicator of the measure of the body's antiaging. T-AOC, as the name describes, is the total capacity of the enzymatic antioxidant system and the nonenzymatic antioxidant system [29]. The antioxidant capacity of the human body's defense system has a close relationship with the health [30].

As previously noted, free radicals generated from D-gal metabolism* in vivo* could be expected to result in aging. It suggests that a substance with excellent free radical scavenging activity has a good antiaging activity. ORAC is a measure of the ability of a substance, especially in the blood, to absorb free radicals utilized in determining the antioxidant effects of foods. The more the content of ORAC is, the stronger the free radical scavenging activity of the food is [2]. The ORAC of EAE was excellent, which matched with its excellent antioxidant and antiaging activity.

The model groups in all experiments showed obvious differences with other groups in daily behavior, pathological sections, and biochemical indexes. In addition, no mouse died due to the model building by D-gal. Therefore, aging models were made successfully. According to our researches, the orally administrated EAE could effectively improve the T-AOC of the antioxidant defense system of the aging mice, enhance the activities of SOD, CAT, and GSH-Px of tissues and serum, increase GSH content, and decrease the MDA content. Meanwhile, the orally administrated EAE could effectively improve the morphologic damage in brain, liver, and kidney induced by D-gal. In a new concept, such nutricosmetics are also known as “beauty pills,” “beauty from within,” and even “oral cosmetics” [31]. Similarly, EAE treated by oral administration could provide visible improvements in skin condition of the aging mice, such as the increase of the Hyp content and SOD activity. Although it was well known that the VC had greater antioxidant activity* in vitro* and* in vivo*, EAE had similar antioxidant activity as shown in our experiments.

Damage resulted from ROS could lead to melanocytic overproduction and to the stepped up consumption of elastin and collagen in the skin [32]. These damages also slow skin's renewability [27]. The accumulative effect is skin wrinkling, fragility, dull appearance, mottled brown pigmentation, and distinct dark spots. Oral and topical antioxidant supplementation serves to augment the body's natural supply of antioxidants and is not meant to completely eradicate free radical production. Rather, it is served to prevent excess damage by enhancing the body's natural defense [2].

Collagen fiber (mainly collagen) is an important component of the dermis. The content of collagen fiber will decrease significantly with the increase of age and thus make the skin saggy and inflexible. Therefore, the change of collagen content can prompt the degree of skin aging. Intradermal Hyp content is rich, which can directly reflect the quantity variation of collagen fiber and thus reflect the aging level of the dermis [6]. Hydroxyproline is accounting for 13.4% of the content of total amino acids in collagen, a minute quantity in elastin and does not exist in other proteins [33]. Hyp content of the skin is seen as an indicator of the level of skin aging [34]. Therefore, many people unilaterally believe that the key to keep skin youthful is to replenish collagen, but actually, elastin is also important. The elasticity and softness of skin are closely linked to elastin. After 25 years old, the human body stops the production of elastin and elastin in the skin will continue to disintegrate and disappear and then the skin begins to sag and fold [35]. Experts point out that women lose elastin faster than men do [32]. This results in an increasing number of women to be concerned about the skin care of antiaging. In addition, the moisture content of the skin is in an important regulatory role in the physiological functions of the skin [6]. The main symptoms of dry skin contain skin tight, peeling, and itching. Therefore, cosmetics with moisturizing property are concerned with the increasing number of women as well [36].

Fortunately, EAE was effective in improving the condition of mice skin, according to our deferring-skin-aging experiment. 1% VE and 4% EAE had similar skin-caring activity. Both of them could effectively enhance SOD activity, maintain collagen, elastin, and moisture content and reduce the MDA content in the aging mouse skin. The anti-skin-aging activity of EAE has been proved vividly by the morphological changes of mouse skin. Particularly, EAE could decrease MDA content effectively in both of the two experiments, which might illustrate EAE had an excellent antilipid peroxidation activity. Our data suggested that EAE delays organism and skin aging via strengthening the antioxidant capacity to some extent. Furthermore, its effect was not less than that of the well-known VC and VE. These results seem to be implying that* Idesia polycarpa* has great development potential of antiaging medicine and skin care product.

## Figures and Tables

**Table 1:** Organ index of mice in each group (mg/g).

Group	Brain	Liver	Kidney
Model	10.64 ± 0.77	38.57 ± 4.21	7.09 ± 0.56
Control	13.07 ± 0.46^b^	47.15 ± 4.63^b^	8.77 ± 0.95^b^
VC	12.15 ± 0.71^b#^	45.37 ± 4.39^b^	7.67 ± 0.80^a#^
Low dose	11.21 ± 0.82^B##^	40.13 ± 3.47^A##^	7.26 ± 0.71^##^
Medium dose	11.95 ± 1.17^a#^	44.80 ± 5.24^a^	8.05 ± 0.75^a^
High dose	12.54 ± 0.83^b^	45.23 ± 2.97^b^	8.97 ± 0.71^bA^

Values are the mean ± SD.

^a^ *P* < 0.05; ^b^ *P* < 0.01 compared with model group (all groups).

^A^ *P* < 0.05; ^B^ *P* < 0.01 compared with VE group (EAE groups).

^#^ *P* < 0.05; ^##^ *P* < 0.01 compared with control group (VC and EAE groups).

**Table 2:** Antioxidants status in the tissue and blood of mice in each group.

Index	Group^C^	Brain	Liver	Kidney	Serum
MDA (nmol/mg prot or nmol/mL)	Model	7.09 ± 1.27	4.31 ± 1.10	5.07 ± 0.71	7.07 ± 0.58
	Control	5.47 ± 0.62^a^	2.23 ± 0.97^a^	3.89 ± 0.51^a^	5.21 ± 0.58^a^
	VC	4.87 ± 0.94^b^	2.27 ± 0.36^b^	3.80 ± 0.68^a^	4.92 ± 0.22^a^
	Low dose	5.53 ± 0.39^a^	3.21 ± 1.15	4.76 ± 0.77	5.11 ± 0.70^a^
	Medium dose	4.77 ± 0.51^b#^	2.57 ± 0.48^a^	3.61 ± 0.80^b^	4.73 ± 0.20^a^
	High dose	4.16 ± 0.50^b##^	2.03 ± 0.63^b^	2.87 ± 0.53^bA#^	5.44 ± 0.63^a^

SOD (U/mg prot or U/mL)	Model	139.29 ± 17.74	281.25 ± 31.04	153.42 ± 5.21	96.64 ± 4.39
	Control	211.64 ± 38.02^b^	368.10 ± 35.78^b^	195.91 ± 16.30^b^	121.70 ± 9.45^a^
	VC	181.12 ± 29.52^b^	384.02 ± 27.66^b^	145.91 ± 16.87^##^	131.65 ± 13.05^a^
	Low dose	158.62 ± 21.84^a#^	349.21 ± 34.90^aA^	171.10 ± 14.59^aA#^	96.41 ± 10.65^A^
	Medium dose	167.80 ± 33.06^a#^	353.29 ± 22.14^bA^	184.68 ± 13.35^bB^	102.26 ± 5.46^A#^
	High dose	175.24 ± 22.22^b#^	320.61 ± 35.27^aB#^	189.27 ± 14.75^bB^	115.62 ± 8.24^a^

GSH (mgGSH/g prot or mgGSH/L)	Model	2.96 ± 0.70	1.10 ± 0.20	1.52 ± 0.25	6.81 ± 0.58
	Control	5.59 ± 0.86^b^	1.72 ± 0.10^b^	2.53 ± 0.16^b^	12.56 ± 0.40^b^
	VC	3.93 ± 0.45^a##^	1.59 ± 0.19^a^	2.29 ± 0.26^b^	7.68 ± 0.38^##^
	Low dose	4.52 ± 0.86^a#^	1.30 ± 0.33^#^	1.63 ± 0.35^B##^	7.62 ± 0.34^##^
	Medium dose	5.83 ± 0.66^bB^	1.56 ± 0.35^b^	1.99 ± 0.29^b#^	7.98 ± 0.21^a##^
	High dose	7.09 ± 0.98^bB#^	2.32 ± 0.33^bB##^	2.52 ± 0.33^b^	9.17 ± 0.42^bA##^

GSH-Px (U/mg prot or U/mL)	Model	×	415.35 ± 47.62	114.84 ± 8.83	459.67 ± 27.24
	Control		657.42 ± 53.53^b^	174.17 ± 11.01^b^	680.33 ± 21.51^b^
	VC		511.67 ± 23.93^b##^	159.10 ± 16.63^b^	626.41 ± 16.88^b#^
	Low dose		430.81 ± 42.50^B##^	151.54 ± 21.95^b#^	508.21 ± 20.52^aB##^
	Medium dose		602.80 ± 67.82^bB#^	169.50 ± 16.33^b^	650.73 ± 14.10^bA^
	High dose		493.69 ± 34.11^b##^	194.91 ± 20.63^bB#^	576.38 ± 14.50^bA##^

CAT (U/g prot or U/mL)	Model	×	295.67 ± 36.16	120.01 ± 13.85	5.42 ± 0.29
	Control		340.24 ± 34.51^a^	166.53 ± 34.24^b^	9.71 ± 0.70^b^
	VC		394.63 ± 62.56^b^	149.67 ± 29.17^a^	12.91 ± 1.19^b^
	Low dose		317.88 ± 22.79^A^	148.28 ± 24.34^a^	8.61 ± 0.47^bB^
	Medium dose		403.77 ± 41.93^b#^	233.29 ± 42.72^bB#^	9.67 ± 0.20^bA^
	High dose		522.61 ± 31.60^bB##^	158.70 ± 24.30^a^	11.42 ± 0.27^b#^

T-AOC (U/mg prot or U/mL)	Model	0.83 ± 0.21	1.23 ± 0.10	0.59 ± 0.06	4.56 ± 0.39
	Control	1.41 ± 0.28^b^	1.66 ± 0.17^b^	1.00 ± 0.15^b^	6.52 ± 0.28^b^
	VC	2.48 ± 0.23^b##^	1.36 ± 0.22^#^	0.46 ± 0.08^##^	6.78 ± 0.04^b^
	Low dose	1.38 ± 0.22^bB^	1.34 ± 0.10^#^	1.18 ± 0.08^bB#^	5.38 ± 0.23^aA#^
	Medium dose	1.53 ± 0.24^bB^	1.58 ± 0.16^b^	1.25 ± 0.11^bB#^	5.85 ± 0.26^bA#^
	High dose	1.92 ± 0.15^bB##^	1.88 ± 0.16^bB#^	1.48 ± 0.13^bB##^	6.31 ± 0.30^b^

Values are the mean ± SD.

× means undetected.

^a^ *P* < 0.05; ^b^ *P* < 0.01 compared with model group (all groups). ^A^ *P* < 0.05; ^B^ *P* < 0.01 compared with VC group (EAE groups). ^#^ *P* < 0.05; ^##^ *P* < 0.01 compared with control group (VC and EAE groups). ^ C^VC group (100 mg/kg); low dose EAE group (100 mg/kg); medium dose EAE group (200 mg/kg); high dose EAE group (400 mg/kg).

**Table 3:** Effects of EAE on the SOD activity and MDA, Hyp, Elastin, and moisture content in mice skin.

Group^c^	MDA (nmol/mg prot)	SOD (U/mg prot)	Hyp (mg/g)	Elastin (ng/mL)	Moisture (%)
Model	15.93 ± 4.41	67.69 ± 16.24	1.08 ± 0.16	815.95 ± 52.67	66.63 ± 1.78
Control	8.76 ± 2.82^b^	119.03 ± 13.55^b^	4.89 ± 0.18^b^	920.43 ± 61.73^a^	75.47 ± 2.64^b^
VE	5.48 ± 4.44^b^	97.02 ± 5.78^b#^	3.07 ± 0.30^b##^	937.73 ± 63.53^a^	70.95 ± 1.30^b#^
Low dose	6.95 ± 2.13^b^	66.96 ± 10.31^B##^	2.43 ± 0.16^bB##^	877.48 ± 97.44	68.05 ± 1.44^A##^
Medium dose	6.34 ± 0.80^b#^	84.32 ± 12.39^##^	2.57 ± 0.35^bA##^	892.24 ± 42.27^a^	69.92 ± 1.59^a#^
High dose	5.51 ± 2.67^b#^	99.07 ± 11.74^b#^	3.09 ± 0.27^b##^	921.63 ± 34.24^b^	70.93 ± 6.26

^a^ *P* < 0.05; ^b^ *P* < 0.01 compared with model group (all groups).

^A^ *P* < 0.05; ^B^ *P* < 0.01 compared with VE group (EAE groups).

^#^ *P* < 0.05; ^##^ *P* < 0.01 compared with control group (VC and EAE groups).

^C^VE group (1%); low dose EAE group (1%); medium dose EAE group (2%); and high dose EAE group (4%).

**Table 4:** Effect of EAE on the thickness of epidermis and dermis.

Group^A^	Thickness of epidermis (*μ*m)	Thickness of dermis (*μ*m)
Model	18.88 ± 1.52	442.01 ± 27.33
Control	22.81 ± 0.52^a^	651.05 ± 34.78^a^
VE	21.24 ± 0.69	529.80 ± 29.56^a#^
Low dose	19.18 ± 1.46	422.34 ± 71.08^##^
Medium dose	19.49 ± 0.50^#^	485.34 ± 43.89^#^
High dose	21.17 ± 0.66	556.33 ± 23.34^b#^

^a^ *P* < 0.05; ^b^ *P* < 0.01 compared with model group (all groups).

^#^ *P* < 0.05; ^##^ *P* < 0.01 compared with control group (VC and EAE groups).

^A^VE group (1%); low dose EAE group (1%); medium dose EAE group (2%); and high dose EAE group (4%).
